# Transforming Apple Pomace into Functional Oligosaccharides via Microwave Optimisation

**DOI:** 10.3390/molecules31152654

**Published:** 2026-07-30

**Authors:** Itsasne Beitia, Leyre Sillero, Itziar Egüés, Izaskun Dávila-Rodríguez

**Affiliations:** 1 Biorefinery Processes Research Group, Department of Chemical and Environmental Engineering, University of the Basque Country EHU, Plaza Europa, 1, 20018 San Sebastián, Spain; 2 Biorefinery Processes Research Group, Department of Chemical and Environmental Engineering, University of the Basque Country EHU, Nieves Cano, 12, 01006 Vitoria-Gasteiz, Spain; leyre.sillero@ehu.eus; 3 Biorefinery Processes Research Group, Department of Chemical and Environmental Engineering, University of the Basque Country EHU, Plaza Rafael Moreno “Pitxitxi” 2, 48013 Bilbao, Spain

**Keywords:** microwave-assisted hydrolysis, apple pomace, oligosaccharide, process intensification, biphasic system

## Abstract

Apple pomace is an abundant agro-industrial residue rich in oligosaccharides, whose efficient and sustainable extraction remains challenging. To overcome these limitations, this study delivers a novel biorefinery strategy by coupling ultrasound-assisted extraction pretreatment with optimised microwave-assisted hydrolysis. Through response surface methodology, the optimal microwave hydrolysis extraction conditions were achieved at 20 min and 120 °C. The obtained oligosaccharides presented a molecular-weight of 191,071 Da, a polydispersity index of 2.19 and a high content of galacturonyl groups (9.95 g/L) and arabino substituents (7.27 g/L). Furthermore, a biphasic system was applied at the optimal point obtained for the monophasic system in order to examine its potential for selective oligosaccharide fractionation. In this case, the galacturonic acid did not form part of the obtained oligosaccharides, and instead they were predominantly formed by galactose and arabinose (6.55 and 4.48 g/L respectively). Moreover, this biphasic setup yielded fewer polydisperse fractions with a lower content of degradation products. Overall, this work highlights the promise of optimised microwave-assisted hydrolysis as a sustainable strategy for oligosaccharide valorisation from apple pomace while demonstrating that the extension to a biphasic system represents a promising alternative for improving process selectivity.

## 1. Introduction

Apple is one of the most widely consumed fruits worldwide, and apple pomace (AP) is the main by-product generated during its industrial processing [1]. This waste accounts for approximately 25–35% of initial apple fruit mass [2], and it is mainly composed of seeds, pulp and peels [3]. In fact, AP is a valuable source of bioactive compounds and essential nutrients, including minerals, dietary fibre, antioxidants, essential fatty acids, and proteins, suggesting its potential for integration into health-promoting food products [4]. Structurally, the insoluble fraction of AP is mainly composed of cellulose, hemicellulose and lignin [5], while the soluble fraction contains pectin and simple sugars including glucose, fructose and galactose, xylose and galacturonic acid [6].

In AP, carbohydrates are the most abundant components [7], including mono-, oligo- and polysaccharides [8]. The bioactivity of oligosaccharides (OS) has been widely documented in the literature, including anti-inflammatory, antioxidant, antibacterial, antitumour and neuroprotective effects [9,10,11,12]. These compounds also act as prebiotics, immune modulators and signalling molecules, playing a key role in gut health, pathogen defence and cellular communication [13,14,15]. Consequently, OS derived from AP are increasingly being explored for innovative uses in health-related and food-based technologies [16].

To isolate bioactive compounds, conventional techniques such as Soxhlet extraction, maceration, hydrodistillation [17] or hydrothermal extraction under reflux heating, among others, are routinely employed [18]. However, these methods suffer from limitations including extended processing times, potential thermal degradation of sensitive compounds, and the need for substantial organic solvent volumes [19].

Compared to conventional extraction methods, green technologies offer significant benefits such as faster processing, more efficient heat and mass transfer, higher product quality, and reduced environmental footprint [20]. Among them, Microwave-Assisted Extraction (MAE) stands out as an environmentally sustainable method that requires shorter processing times, consumes less solvent and energy, and achieves higher extraction yields [21]. This enhanced efficiency is mainly attributed to rapid internal heating of water and improved mass transfer occurring within a shorter time frame [21]. Consequently, MAE has been widely employed in the extraction of a wide range of bioactive compounds [22], including OS [23]. For instance, Pérez-Pérez et al. (2023) [24] demonstrated the successful recovery of OS from *Robinia pseudoacacia* wood, highlighting MAE as an effective and eco-friendly pretreatment for biorefinery applications. Similarly, Torrado et al. (2023) [25] successfully produced xylooligosaccharides from pine nut shells through microwave-assisted autohydrolysis, using water as a solvent, confirming the efficiency and sustainability of this approach for carbohydrate valorisation.

Ultrasound-Assisted Extraction (UAE) has also been widely applied as green technology for recovering polyphenols, polysaccharides, carotenoids and other bioactive compounds from fruit and plant matrices, as it operates at low temperatures and requires short extraction times with minimal energy and solvent consumption [26]. UAE relies on the formation and collapse of cavitation bubbles generated as ultrasonic waves propagate through the extraction medium, promoting cell wall disruption and enhancing mass transfer. Nour et al. (2021) [23] demonstrated that both MAE and UAE can release oligosaccharides under controlled conditions, emphasizing the ability of ultrasound to selectively disrupt hemicellulosic linkages and promote the solubilisation of short-chain carbohydrates. However, most studies have focused on antioxidant-rich extracts, and research specifically addressing UAE for OS release is largely lacking. While simultaneous ultrasound–microwave systems have been more explored for various biomass applications [27], the evaluation of sequential UAE followed by microwave-assisted hydrolysis (MAH) for OS extraction remains largely unexplored.

A promising approach to implementing this sequential strategy involves the use of biphasic reaction systems, due to their ability to simplify processing steps and enhance overall reaction yields [28]. A biphasic system consists of a hydrophilic phase and a hydrophobic phase, typically water and an organic solvent [7]. Biphasic systems offer several advantages, as they allow thermodynamic control and minimise side reactions [28]. Short-chain alcohols, such as n-butanol are commonly used for biomass pretreatment, particularly for the selective extraction of lignin and hemicellulose [29]. In water/n-butanol biphasic systems, the solvent’s recyclability, low toxicity, and cost-effectiveness make it a promising green option for large-scale applications [30].

**Figure 1:**
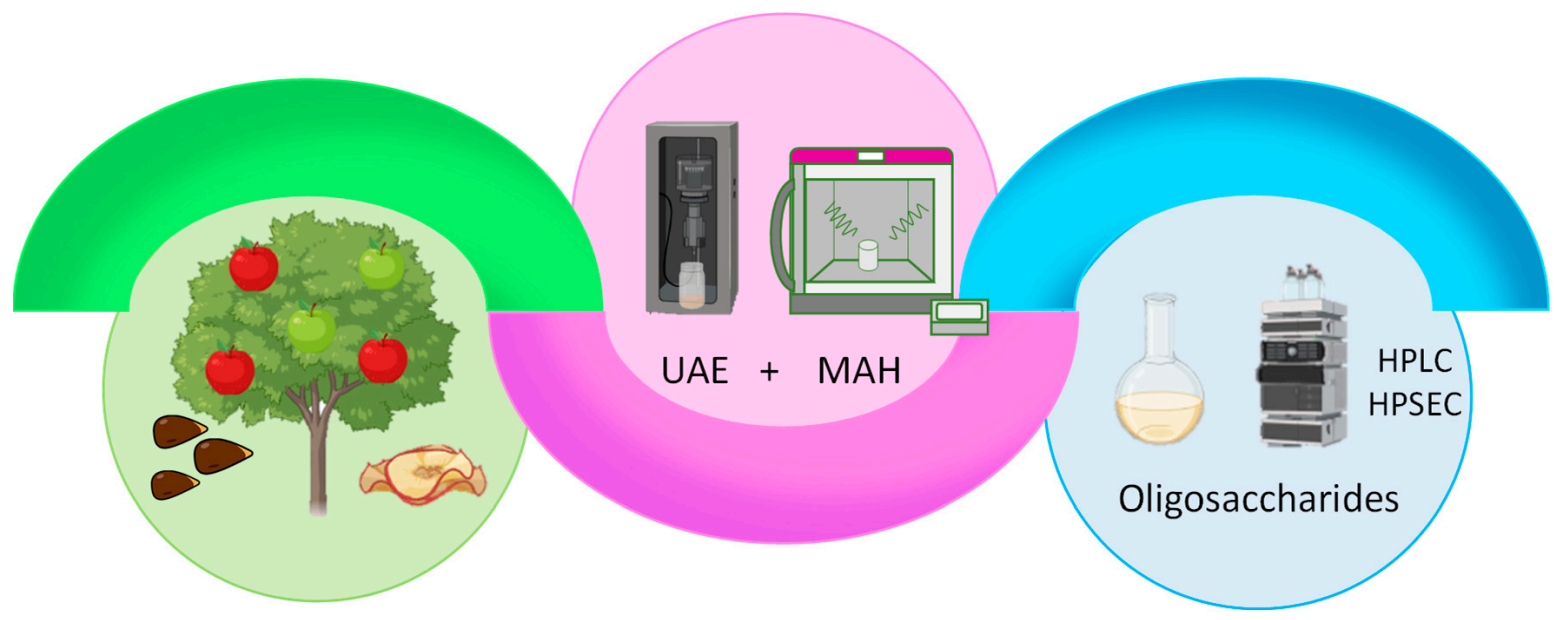
Schematic representation of oligosaccharide extraction.

In this context, the main objective of the present work was to advance the valorisation of AP by developing a sequential UAE–MAH process, focusing specifically on the optimisation of the sequential MAH step. First UAE was applied as a pretreatment to ensure the removal of non-structural sugars. Then a systematic evaluation of the MAH stage was carried out, (studying the reaction time, temperature, and acid concentration) focusing on its ability to maximise OS production while minimising monosaccharide release. The optimal point was subsequently validated experimentally, and the resulting liquors from the optimal conditions were characterised by HPLC and HPSEC to determine the chemical composition and molecular weight of the oligosaccharides that they contained, as it can be seen in Figure 1. Finally, a biphasic water/n-butanol system was tested, under the optimal hydrolysis conditions obtained for the monophasic system, in order to examine its capacity for selective recovery and cleaner separation of OS.

## 2. Results and Discussion

### 2.1. Composition of the Raw Material

The AP used in this study presented the following chemical composition on a dry-weight basis: Among the carbohydrates present in the AP, glucose was the predominant component, accounting for 32.15 ± 1.58% of the dry raw material, followed by xylose, 3.68 ± 0.40; arabinose, 3.89 ± 0.61; galactose, 4.56 ± 0.63; fructose, 8.72 ± 0.23 (the fructose content can be attributed to incomplete removal of residual apple juice during the washing step); galacturonic acid, 6.65 ± 0.40; and acetic acid, 1.55 ± 0.12. Lignin content was 27.73 ± 2.26%, while smaller fractions of extractives (14.10 ± 1.21%) and ashes (0.72 ± 0.05%) were also detected. Depending on the origin of the apple pomace and the processing method some variations can be observed on its composition. However, the content of the major component is consistent with the results obtained by Melikoğlu et al. (2019) [31], who documented glucan content around 32%, corresponding exclusively to glucose, and Lozano et al. (2026) [32], who found a glucan content of 29% also corresponding to glucose, which represented the predominant carbohydrate fraction. Regarding the composition of the carbohydrates, the results obtained align with the ones obtained by Mehrländer et al. (2002), who reported obtaining arabinans, xyloglucans, rhamnogalacturonans and arabinogalactans during the enzymatic liquefaction of apple pomace [33]. The high carbohydrate content further supports the suitability of this residue for microwave-assisted hydrolysis, as it provides an abundant and readily convertible source of OS.

### 2.2. Experimental Design Optimisation

Given the interest in selectively recovering OS due to their well-known properties and the need to identify the conditions that maximise their production, an optimisation study was carried out to evaluate the influence of the main reaction variables on the process. The results obtained from the experimental design are summarised in Table 1.

The OS concentration obtained across the experimental conditions ranged between 0 and 27.85 g/L. The minimum value was recorded at the highest reaction temperature, shortest reaction time and intermediate catalyst concentration, indicating that excessive thermal severity coupled with insufficient reaction time prevents OS accumulation. That lack of OS acquisition can be associated with more severe processing conditions, which promote depolymerisation of the potential OS. Moreover, the experiments conducted at the maximum catalyst concentration consistently showed reduced OS yields, which could promote further depolymerisation of the oligosaccharides formed, ultimately leading to their fragmentation into monosaccharides. By contrast, maximum OS was obtained under mild–moderate conditions, low catalyst concentrations, short reaction times and intermediate reaction temperatures, which favoured controlled hydrolysis without excessive chain cleavage.

In the case of monosaccharides, concentrations ranged from 9.68 to 41.07 g/L. The minimum value was obtained under the mildest conditions, in the absence of a catalyst, with the shortest reaction time and an intermediate temperature, which limited depolymerisation and therefore reduced the formation of monomeric sugars. By contrast, the highest monosaccharide concentration was obtained under more severe conditions, combining longer reaction times with higher temperature and 0.025 M catalyst concentration, which favoured the breakdown of OS into their monomeric sugars. Taken together, the opposing trends observed for OS and monosaccharides confirm that mild–moderate severities favour controlled hydrolysis and OS accumulation, whereas harsher conditions enhance depolymerisation, shifting the product distribution towards monosaccharides.

An analysis of variance (ANOVA) was performed to evaluate the fit quality and the statistical relevance of the factors and model terms for both responses. Table 2 presents the analysis of variance (ANOVA) for the fitted response surface models, providing a detailed assessment of their statistical significance, adequacy, and goodness of fit. The coefficient of determination (R^2^) for oligosaccharides was 0.946, indicating that 94.6% of the variability in OS yield is explained by the model. At a 95% confidence level, the linear effects of temperature (*x*_1_) and catalyst concentration (*x*_3_), as well as the quadratic term of catalyst concentration (*x*_3_^2^), were statistically significant. These results corroborate the process trends discussed above: temperature and acid loading exert first-order control on severity, while the curvature in *x*_3_ captures the over activation regime that depresses oligosaccharide accumulation. The fitted second-order polynomial equation (Equation (1)) is as follows:

(1) $$y\;\left(g/L\right)=2.3-7.95x_{1}-0.24x_{2}-5.19x_{3}+2.52x_{1}^{2}+2.19x_{1}x_{2}+2.88x_{1}x_{3}+4.87x_{2}^{2}+3.92x_{2}x_{3}+10.10x_{3}^{2}$$

In the case of monosaccharides, the model achieved an R^2^ of 0.868. Within this model, the quadratic term of catalyst concentration (*x*_3_^2^) was statistically significant (*p* < 0.05), reflecting the non-linear escalation of depolymerisation at higher acid levels. The corresponding second-order polynomial equation (Equation (2)) is presented below:

(2) $$y\;\left(g/L\right)=36.39+1.87x_{1}+0.06x_{2}+4.19x_{3}-7.48x_{1}^{2}-4.66x_{1}x_{2}-5.23x_{1}x_{3}-5.28x_{2}^{2}-2.10x_{2}x_{3}-10.78x_{3}^{2}$$

Together, these ANOVA findings are consistent with the observed product distributions: moderate severities (controlled temperature and zero catalyst) favour OS accumulation, whereas higher amounts of catalyst, represented by the significant *x*_3_^2^ term, drive excessive depolymerisation, shifting the system toward monosaccharide formation.

#### 2.2.1. Evaluation of the Influence of the Variables in Oligosaccharide Content

**Figure 2:**
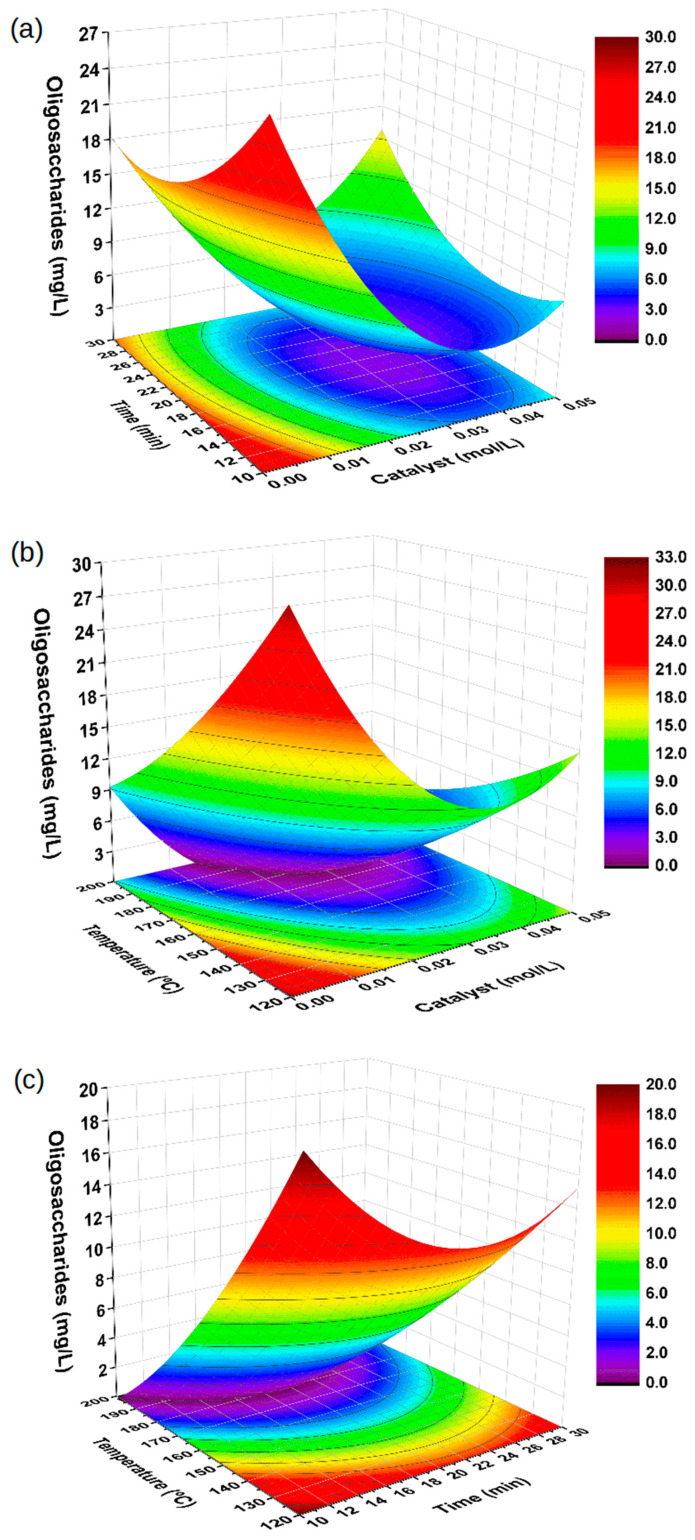
Oligosaccharide concentration fixing (**a**) temperature, (**b**) time and (**c**) catalyst concentration at their middle levels (x = 0).

Figure 2a illustrates the interaction between reaction time and catalyst concentration at the central temperature level (*x*_1_ = 0). At this fixed temperature, increasing acid concentration negatively affects OS yield across the entire time range, while reaction time exhibits a non-linear effect, indicating the existence of an optimal time window under mild catalytic conditions. These trends are consistent with the statistical model, which reflects the combined influence of these variables and the presence of optimal conditions for oligosaccharide formation. A similar tendency has been reported by Torrado et al. (2024) [25] who observed that above optimal conditions, when severity is increased oligosaccharide levels decline due to their conversion to monosaccharides and degradation products (furfural and 5-hydroxymethylfurfural). This is consistent with the behaviour observed in the present work.

Figure 2b displays a model-derived response surface illustrating the interaction effects of temperature and H_2_SO_4_ concentration on oligosaccharide yield at a fixed reaction time (*x*_2_ = 0). The results reveal that lower temperatures and lower acid concentrations lead to higher OS yields, whereas harsher conditions lead to a pronounced decrease in yield. This behaviour suggests that excessive thermal and acidic severity promotes secondary reactions, such as further hydrolysis and degradation, thereby reducing OS yield. This trend is well captured by the statistical model, which highlights the increasing relevance of combined effects under more severe conditions. These observations are consistent with the results reported by Hao et al. (2025) [34]. In that work, Hao and Zhang (2025) [34] optimised the extraction of OS from *Hedysarum polybotys*, obtaining the same trend regarding the inverse relationship between temperature and OS yield. However, the optimal extraction temperatures reported in their study (68 °C) were lower than those identified in the present work.

Figure 2c presents the combined influence of reaction time and temperature at the central catalyst level (*x*_3_ = 0). The response surface reveals that higher temperatures intensify the negative effect of prolonged reaction times, confirming that OS formation is favoured under low–mild thermal conditions and short reaction times. This behaviour is also reflected in the model, which indicates the presence of optimal conditions followed by a decline in yield due to degradation processes. Overall, these results highlight the importance of controlling process severity to maximise OS yield while minimising degradation. This behaviour is consistent with previous findings reported by Eblaghi et al. (2021) [35], who observed that when treatment times exceeded 25 min and temperatures rose above 120 °C, extensive depolymerisation occurred, leading to a marked decline in the solubilised carbohydrate fraction, an effect fully aligned with the decrease in oligosaccharides observed under severe conditions in the present study.

#### 2.2.2. Evaluation of the Influence of the Variables in Monosaccharide Content

**Figure 3:**
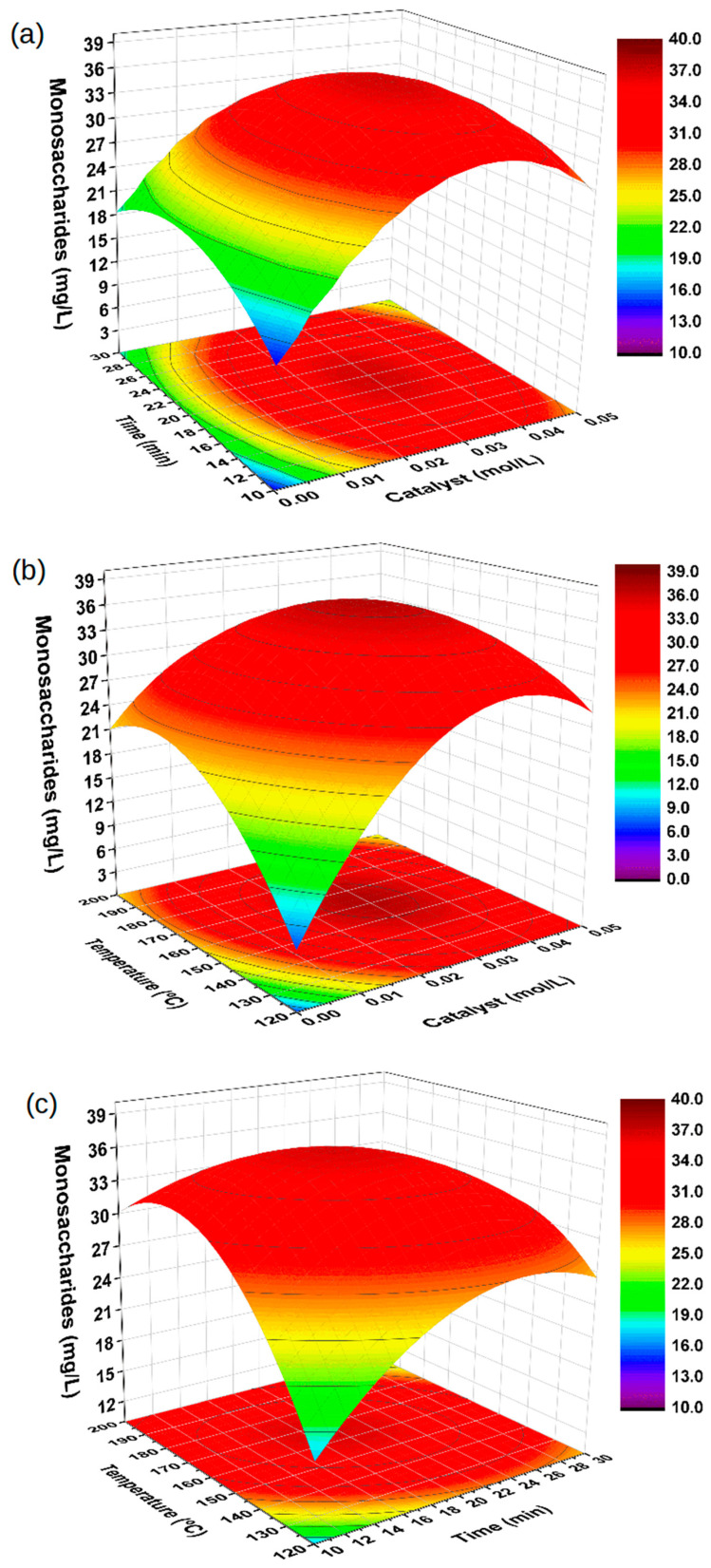
Monosaccharide concentration fixing (**a**) temperature, (**b**) time and (**c**) catalyst concentration at their middle levels (x = 0).

Figure 3a illustrates the interaction between reaction time and catalyst concentration at the central temperature level (*x*_1_ = 0). The results reveal that monosaccharide concentration depends on both time and catalyst concentration. As the reaction time increases, the monosaccharide yield rises, but beyond a certain reaction duration, degradation becomes predominant and the concentration declines. A similar trend is observed for the catalyst concentration: These trends are consistent with the statistical model, where x_1_ and x_3_, as well as their interaction, present notable effects (Table 2), confirming their combined influence on sugar release and degradation.

Figure 3b displays the model-derived response surface illustrating the interaction effects of temperature and H_2_SO_4_ concentration on monosaccharide yield at a fixed reaction time (*x*_2_ = 0). At low temperatures, the polysaccharide breakdown is limited, leading to low monosaccharide production. When temperature increases, monosaccharide yield increases and excessive temperatures promote degradation reactions. This behaviour corroborates the model, where temperature (*x*_1_) shows a lower individual effect compared to x_3_ and quadratic terms, indicating that its influence becomes more relevant in combination with other factors and at higher severities.

Figure 3c presents the combined influence of reaction time and temperature at the central catalyst level (*x*_3_ = 0). Monosaccharide release increases with both variables up to optimal values, after which degradation dominates. This behaviour is supported by the significance of quadratic terms (*x*_1_^2^ and *x*_3_^2^) and their negative coefficients, which indicate the presence of optimal conditions followed by a decline due to degradation phenomena. A similar trend is observed with reaction time, where short times favour monosaccharide formation and long times lead to product degradation. The behaviour observed in the present work for monosaccharide concentration is in accordance with the results reported by Zong et al. (2024) [36], who similarly reported that increases in temperature, reaction time, and acid concentration follow the same pattern: enhanced monosaccharide release at mild severities followed by rapid degradation once optimal conditions are exceeded. A similar trend was found by Pérez-Pérez et al. (2023) [24] where monosaccharides such as xylose increase to an optimum point but decrease with process severity (temperature and time).

#### 2.2.3. Model Validation

The mathematical model also enabled the optimisation of the treatment conditions to maximise the production of OS and minimise the production of monosaccharides. According to the model, the optimal treatment temperature was 120 °C, the optimal MAH duration was 20 min, and the optimal concentration corresponded to the absence of H_2_SO_4_, predicting an OS yield of 30.95 g/L. Experimental validation of these conditions produced an OS concentration of 28.25 ± 3.34 g/L, in close agreement with the model prediction. This consistency confirms the robustness and predictive capability of the optimisation approach for OS production.

### 2.3. Product Characterisation

#### 2.3.1. Chemical Characterisation

The principal OS found in the aqueous phase included arabino substituents (ArOS) 7.269 ± 1.987 g/L, galactooligosaccharides (GalacOS) 5.563 ± 2.264 g/L, glucooligosaccharides (GOS) 3.765 ± 1.922 g/L, xylooligosaccharides (XOS) 0.899 ± 0.002 g/L, acetyl substituents (AcOS) 0.807 ± 0.093 g/L and galacturonyl groups (GalAOS) 9.951 ± 2.926 g/L. Regarding free monosaccharides, the concentrations measured were as follows: fructose (0.872 ± 0.601 g/L), followed by glucose (0.295 ± 0.117 g/L), arabinose (0.162 ± 0.003 g/L), galactose (0.049 ± 0.004) g/L and xylose (0.044 ± 0.044 g/L). Degradation products were also detected, reaching concentrations of 0.054 ± 0.016 g/L. Representative HPLC chromatograms of the mixed standards and characteristic samples are provided in the Supplementary Material (Figure S1).

#### 2.3.2. Molecular-Weight Distribution

The chromatographic profile after HPSEC revealed two distinct molecular weight populations of solubilised carbohydrates: a predominant medium-molecular-weight fraction (±10,000 Da; 65.77%) and high-molecular-weight fraction (±100,000 Da; 34.23%). The high-molecular-weight population which exhibited an average molecular-weight (Mw) of 191,071 Da with a relatively narrow polydispersity index (PDI = Mw/Mn = 2.19). Given this high-molecular-weight, this population is far beyond the conventional range of oligosaccharides and is instead classified as a water soluble, partially depolymerized polysaccharide fraction.

In contrast, the medium-molecular-weight population showed an Mw of 10,535 Da and a broader PDI = 6.52, indicating a more heterogeneous distribution of chain length. This broader distribution is consistent with the progressive, non-uniform cleavage of carbohydrate structures during hydrolysis, where partial depolymerisation generates a wide spectrum of oligosaccharide sizes. The chromatograms of the molecular weight distributions are shown in Figure S2.

#### 2.3.3. Solid-Phase Characterisation

The solid phase remaining after the monophasic MAH exhibited 34.9% solubilisation of the biomass, which is in line with previous aqueous extraction yields reported for AP such as the ±30% water-soluble fraction obtained by Fernandes et al. (2019) [37]. Compositional analysis of the residual solid revealed that the process selectively removed 49.7% of the carbohydrates present in the apple pomace, while lignin remained unaltered, showing only a minimal loss of 2.6%. This limited impact on the lignin fraction highlights the effectiveness of hydrolysis as a mild, carbohydrate-targeted pretreatment, preserving the integrity of the lignin matrix and thereby facilitating its subsequent removal in later delignification stages [38].

### 2.4. Evaluation of a Biphasic System Under Optimum Conditions Determined for the Monophasic System

#### 2.4.1. Chemical Characterisation

**Figure 4:**
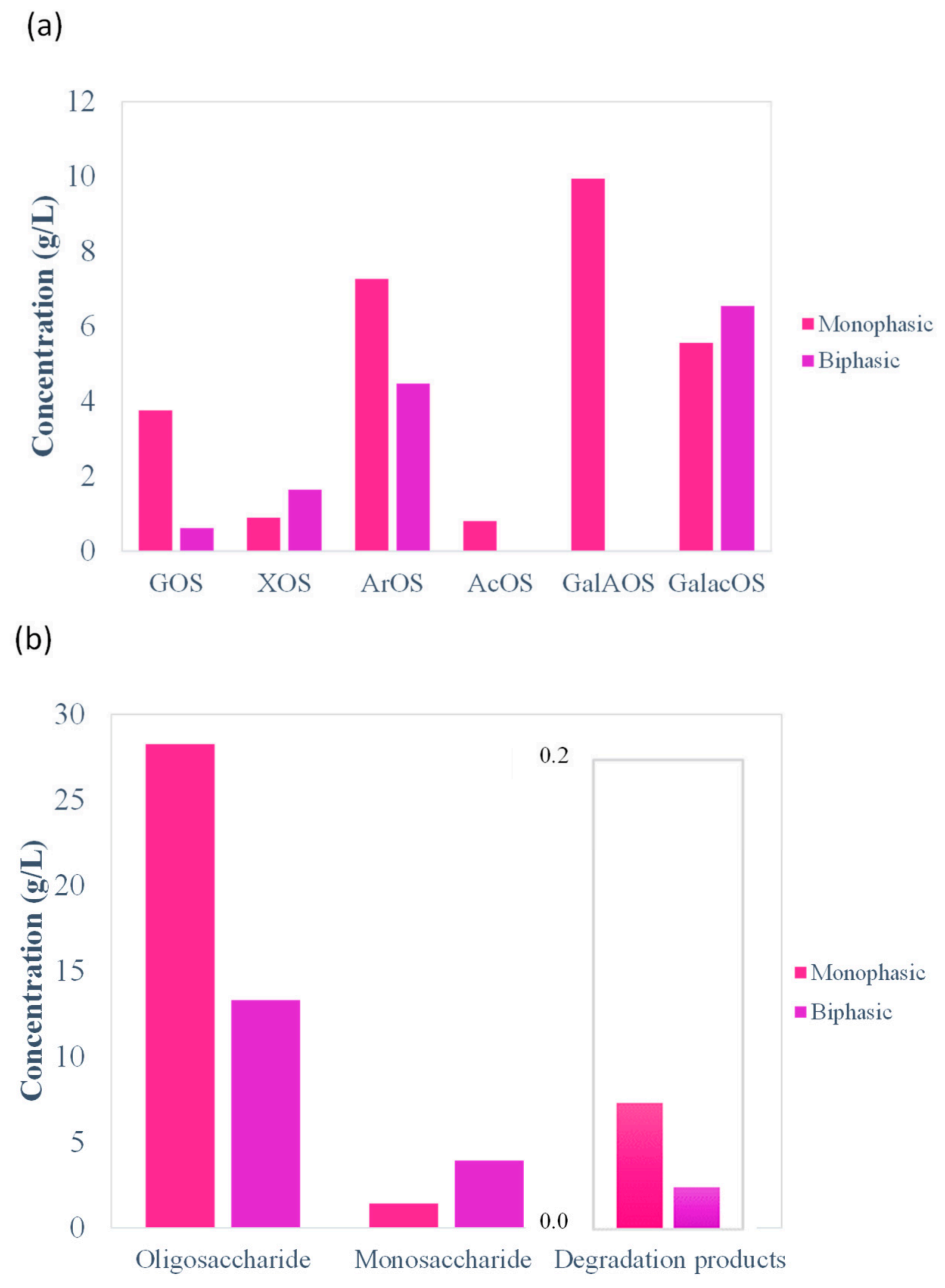
Compositions obtained after MAH with monophasic and biphasic systems for (**a**) distribution of individual oligosaccharides and (**b**) global profile including oligosaccharides, monosaccharides and degradation products.

The OS profiles obtained under monophasic and biphasic MAH conditions showed marked differences in both abundance and structural distribution (Figure 4). As shown in Figure 4a, the monophasic system generated a broader OS spectrum, with the notable presence of GalAOS. In addition to GalAOS, ArOS and GalacOS also appeared at comparatively higher concentrations after the monophasic treatment. In contrast, the biphasic system produced a markedly different composition: The predominant compounds were GalacOS (6.547 ± 2.424 g/L) and ArOS (4.480 ± 0.656 g/L), while neither AcOS nor GalAOS were detected in the biphasic system. The variations observed in the composition of the oligosaccharides could be attributed to the addition of n-butanol to the reaction media.

Moreover, apart from the composition of the oligosaccharides, the amount of oligosaccharide, monosaccharide and degradation by-products obtained when the MAH was carried out using a monophasic or a biphasic system was different, as can be seen in Figure 4b. The monophasic system yielded a higher concentration of OS compared to the biphasic system. This result was contrary to what it was expected since biphasic systems can act as catalytic facilitators by promoting molecular interactions at the liquid–liquid interface, which significantly accelerates reaction kinetics. This phase-transfer approach optimises the process by continuously removing inhibitory by-products from the reaction site, shifting the thermodynamic equilibrium to favour the formation of target compounds [39].

However, in this case the use of the biphasic system caused the opposite effect, which could be related to the inherent characteristics of the alcohol used, for instance its steric hindrance effect, polarity, dielectric loss and dielectric constant. Liang et al. (2024) suggested that the use of an alcohol as the organic solvent of a biphasic system could reduce the degradation process of the monosaccharides [40]. Thus, the addition of the n-butanol, instead of presenting a catalytic effect, due to the removal of the degradation product formed from the monosaccharides, which shifts the chemical equilibrium toward the formation of products, could cause the opposite effect. Since the reaction when the biphasic system was used was less effective than when the monophasic system was used, it might have only enough severity to remove the pyranoses of the side chains of the polysaccharide, which are the most susceptible to acid hydrolysis [41].

Although the quantity of oligosaccharides obtained after carrying out the reaction in a biphasic system was lower than when a monophasic system was used, the obtained OS were purer due to the reduced presence of degradation products in the reaction media.

#### 2.4.2. Molecular-Weight Distribution

**Figure 5:**
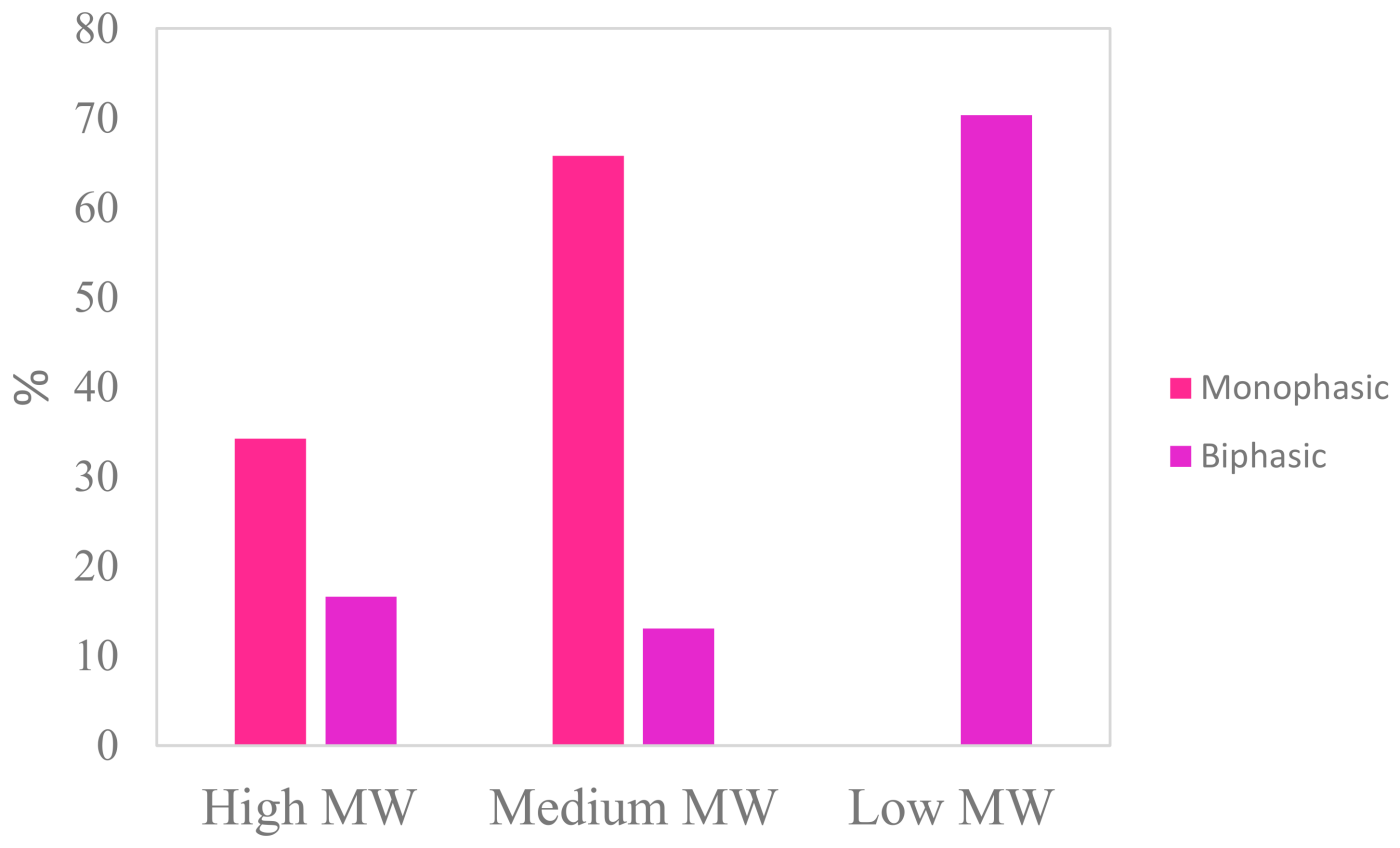
Molecular-weight distributions of monophasic and biphasic systems.

Comparison of the HPSEC chromatographic analysis revealed different molecular weight distribution profiles in the water phase between monophasic and biphasic MAH systems (Figure 5).

Consistent with the higher oligosaccharide yields and the lower monosaccharide content observed under monophasic conditions, this system predominantly generated medium- and high-molecular-weight populations, reflecting a controlled depolymerisation process and the limited formation of low-molecular-weight species. In contrast, biphasic extraction produced a markedly more diversified profile dominated by low-molecular-weight compounds (±200 Da; 70.35%), accompanied by smaller proportions of medium- (13.05%) and high-molecular-weight (16.61%) fractions than the ones found in the monophasic system. The high-molecular-weight population of the biphasic system presented an Mw of 190,142 Da with a PDI of 3.21, so it was attributed to partially depolymerised polysaccharides, whereas the medium-molecular-weight population exhibited an Mw of 3108 Da and a PDI of 1.43; the low-molecular-weight fraction displayed an Mw of 173 Da and a PDI of 2.10. The higher percentage of low-molecular-weight compounds obtained during the MAH carried out with the biphasic system was in agreement with the results obtained during the chemical characterisation of the aqueous phase obtained after the MAH. Thus, the low-molecular-weight compounds determined in the aqueous phase obtained after the treatment carried out with the biphasic system could be attributed to the removal of the side chains of the polysaccharides [41].

Overall, the HPSEC data reinforce that monophasic MAH favours the recovery of structurally diverse, medium-sized oligosaccharides, whereas the biphasic system shifts the product spectrum toward smaller, more uniform carbohydrate species.

#### 2.4.3. Solid-Phase Characterisation

When comparing the solid fraction obtained after monophasic and biphasic MAH treatments, both treatments resulted in similar global solubilisation levels (34.9% and 33.6%, respectively). However, their compositional profiles revealed markedly different behaviours. The biphasic system exhibited different selectivity, characterised by extensive lignin removal (53.9%) but considerably lower solubilisation of carbohydrate (24.4%) than the monophasic system. Other studies, however, report significantly higher carbohydrate solubilisation, mainly attributed to the hemicellulosic fraction, under different biphasic conditions. For example, Renders et al. (2018) [42] reported up to 85% hemicellulose solubilisation in *Eucalyptus sawdust* using a water/n-butanol biphasic system under catalytic conditions. Schmetz et al. (2019) [43] demonstrated that the H_2_SO_4_/n-butanol biphasic system increased xylan-to-xylose conversion from 55% to 73% in sugar cane bagasse. Together, these findings suggest that the biphasic system employed in this study or the employed reaction conditions were not optimal. Nevertheless, biphasic approaches remain promising provided that the operational parameters are appropriately optimised.

## 3. Materials and Methods

### 3.1. Raw Material Characterisation

AP was supplied by Olagi cider house *(Altzaga, Basque Country, Spain).* Upon receipt, and in order to avoid putrefaction, AP was stored at −18 °C. Prior to its use, the feedstock was thawed in an oven at 50 °C for 72 h, with occasional stirring to ensure uniformity. Following this, the raw material was ground and sieved to a particle size below 0.5 mm.

First, the characterisation of the AP was conducted to determine the structural components of the AP following TAPPI standards methods. Prior to structural characterisation, the AP was washed as a pretreatment using Soxhlet extraction under reflux conditions to remove the adhered free soluble sugars originating from the residual apple juice following the National Renewable Energy Laboratory (NREL) TP-510-42619 protocol [44]. The remaining solid residue was then dried in the 50 °C oven for 24 h and then characterised to determine its structural compounds. As part of the feedstock characterisation, the extractives content, ash content and moisture were determined according to TAPPI standards T204 cm-97, T211 om-02 and T264-om-88, respectively. Additionally, the concentrations of carbohydrates and acid-insoluble lignin were measured according to the standard protocol TP-510-42618 established by the NREL, employing quantitative acid hydrolysis [45]. This analytical procedure was followed by the method described in (Dávila et al., 2016) [46] and the HPLC analysis of the liquors was performed using the method described in Section 3.4.1 Liquid phase characterisation.

### 3.2. Microwave-Assisted Hydrolysis Optimisation

#### 3.2.1. Microwave-Assisted Hydrolysis Procedure

Prior to the microwave procedure, an UAE pretreatment was carried under conditions previously optimised in a separate study: 66 °C, for 16 min with an amplitude of 80%, applying a solid-to-liquid ratio of 1:10 (*w*/*v*), in order to remove non-structural sugars. The sonication was performed using a Vibra-Cell™ VC 750W Ultrasonic Liquid Processor (Sonics & Materials, Newton, CT, USA) where the AP was subjected to sonication in a container connected to a cooling bath with a water recirculation pump to regulate the ultrasound-induced temperature increase. This step was followed by MAH that was carried out in a closed vessel using a microwave system, operating at 500 W, which monitors the pressure as well as the temperature (MILESTONE flexiWAVE, Shelton, CT, USA). For microwave hydrolysis, AP was mixed with distilled water in digestion vessels at a solid-to-liquid ratio of 1:10 (*w*/*w*). The experiments were conducted at varying reaction times, temperatures, and sulfuric acid concentrations according to the experimental ranges defined in Section 3.2.2 Experimental design. After reacting, samples were filtered to separate the liquid and solid phases. The solid was cleaned with water until a neutral pH and was characterised as described in Section 3.4.1 Liquid phase characterisation. The resulting supernatant was also analysed by HPLC characterisation analyses performed in triplicate.

#### 3.2.2. Experimental Design

In this study, the optimisation strategy focused on maximizing OS yield (Y_1_) while minimising the generation of monosaccharides (Y_2_), thereby enhancing the selectivity of the hydrolysis process. To systematically investigate the influence of the main operational variables on these two response factors, a three-level-three-factor Box–Behnken Design (BBD) was employed. Statistical analysis of the design was carried out using STATGRAPHICS Centurion version XVI software (Statpoint Technologies INC., Warrenton, VA, USA). The selected independent variables were temperature (*x*_1_), time (*x*_2_) and sulphuric acid concentration (*x*_3_). The ranges selected for temperature (120–200 °C), reaction time (10–30 min), and acid catalyst concentration (0–0.05 M) were established based on prior experimental knowledge and preliminary studies. A total of 15 experimental runs were conducted, including 3 replicates in the central point, to estimate experimental error. Once the results from the different experiments were obtained, response surface methodology (RSM) was applied, enabling the construction of predictive models that describe the relationship between the independent variables and the response. These models were used to identify optimal conditions and analyse the significance of each factor. The second-order polynomial equation used for the data was the following Equation (3).

(3) $$y=\beta_{0}+\sum_{i=1}^{3}\beta_{i}x_{i}+\sum \sum_{i<k=1}^{3}\beta_{ik}x_{i}x_{k}+\sum_{i=1}^{3}\beta_{ii}x_{i}^{2}$$

where *y* represents the concentration of oligosaccharides or monosaccharides, considered as the predicted response, *β*_0_, *β*_i_, *β*_ik_ and *β*_ii_ are the regression coefficients from the experimental results and *x*_i_ and *x*_k_ are the normalised and dimensionless independent variables, varying from −1 to 1. The quality of the fitted model was assessed using the coefficient of determination (R^2^), and an analysis of variance (ANOVA) was performed to evaluate statistical significance at a 95% confidence level. The predicted optimal point was experimentally validated by conducting the corresponding experiments by triplicate.

### 3.3. Evaluation of a Biphasic System for the Oligosaccharide Acquisition by MAH

To further enhance selectivity and improve the downstream recovery of OS, a biphasic water/n-butanol extraction system was subsequently evaluated. As this system was not independently optimised, the evaluation was performed as a preliminary study under the optimal hydrolysis conditions obtained for the monophasic system.

After biphasic MAH, the samples were filtered and the two phases were separated by decantation, obtaining an organic phase and an aqueous phase separately. The aqueous phase was recovered for further processing. Samples were characterized as described in Section 3.4 Analytical techniques. Analyses were performed in triplicate.

### 3.4. Analytical Techniques

#### 3.4.1. Liquid Phase Characterisation

Chemical characterisation of the aqueous liquid phases obtained from MAH and biphasic MAH was performed through the quantification of mono- and OS using HPLC and HPSEC, employing a Jasco LC Net II/ACD chromatograph (Jasco Analitica Spain, S.L., Madrid, Spain) equipped with a refractive index detector.

Monosaccharides (glucose, xylose, arabinose, galactose and fructose) were quantified using a CarboSep CHO 682 column (Transgenomic Inc., Omaha, NE, USA) operated with water as mobile phase at 80 °C and a flow rate of 0.4 mL/min. Galacturonic acid, acetic acid and degradation products were quantified on an Aminex HPX-87H column (BioRad Life Science Group, Hercules, CA, USA) using 0.005 M H_2_SO_4_ as the mobile phase at 40 °C and a flow rate of 0.6 mL/min.

To assess the amount of OS obtained, each sample was also subjected to a post-hydrolysis process. The methodology employed for oligosaccharide quantification is based on a comprehensive mass-balance approach suitable for biorefinery screening. While this approach enables an overall assessment of oligosaccharide recovery, it does not provide information on the origin of the monosaccharides determined, thus preventing a detailed structural characterisation of the polymers. In this procedure, concentrated sulphuric acid (96%) was added to the liquid fraction until reaching a final concentration of 4%. The mixture was autoclaved at 121 °C for 30 min to ensure complete breakdown of all oligomeric structures into their constituent monomeric units, then it was cooled and filtered before analysis by HPLC. The concentrations of the individual OS fractions (ArOS, GalacOS, GOS, XOS and GalAOS) were then calculated from the increase in their corresponding free monosaccharides after total acid hydrolysis.

Finally, to deepen the structural assessment of the OS recovered at the optimal point of MAH and of the biphasic MAH, the corresponding liquors were analysed by high-performance size-exclusion chromatography (HPSEC) using Jasco LC Net II/ACD chromatograph (Jasco Analitica Spain, S.L., Madrid, Spain). Analyses were performed on a PL Aquagel-OH column (Agilent Technologies, Inc., Santa Clara, CA, USA) operated at 40 °C, using 0.005 N H_2_SO_4_ as the mobile phase at 0.6 mL/min, which enabled the determination of the molecular-weight distribution of the oligosaccharidic fraction. Calibration curve was made using Pullulan polysaccharides (Agilent Technologies, Inc., Santa Clara, CA, USA) with different molecular weights (between 180 and 805,000 Da).

#### 3.4.2. Solid-Phase Characterisation

The solid phases obtained after MAH were washed with water until a neutral pH, air dried and subjected to moisture determination according to the TAPPI T264-om-88 standard. Quantitative acid hydrolysis of the solids was carried out following the NREL TP-510-42618 protocol mentioned in Section 3.1 Raw material characterisation, in order to determine the carbohydrate and lignin content [45].

## 4. Conclusions

This study successfully optimised MAH treatment for oligosaccharide extraction achieving an efficient recovery. The optimal conditions enabled a selective and controlled autohydrolysis, maximising oligosaccharide formation while limiting undesired depolymerisation. Structural characterisation of the resulting liquors confirmed the formation of oligosaccharides in distinct molecular-weight distributions. While the biphasic setup produced lower OS yields, likely because it was operated under monophasic optimum conditions rather than an independently optimised environment, it nevertheless generated cleaner liquid fractions with reduced degradation products and narrower molecular weight distributions. These results indicate that, once specific process optimisation is carried out, the biphasic approach remains a promising alternative.

## Figures and Tables

**Table 1:** Experimental conditions and results for MAH.

Exp	Temperature, °C (*x*_1_)	Time, min (*x*_2_)	Catalyst, [H_2_SO_4_], M (*x*_3_)	Oligosaccharides (g/L)	Monosaccharides (g/L)
1	200 (1)	10 (−1)	0.025 (0)	0.00	31.49
2	160 (0)	20 (0)	0.025 (0)	2.51	41.07
3	120 (−1)	30 (1)	0.025 (0)	15.02	25.08
4	120 (−1)	10 (−1)	0.025 (0)	22.45	18.36
5	120 (−1)	20 (0)	0.025 (0)	16.23	22.92
6	200 (1)	30 (1)	0.025 (0)	1.31	19.58
7	160 (0)	30 (1)	0 (−1)	22.13	16.85
8	160 (0)	20 (0)	0.025 (0)	2.09	38.90
9	200 (1)	20 (0)	0 (−1)	7.85	23.79
10	120 (−1)	20 (0)	0 (−1)	27.34	9.68
11	160 (0)	20 (0)	0.025 (0)	5.24	29.19
12	160 (0)	30 (1)	0.05 (1)	14.54	26.64
13	200 (1)	20 (0)	0.05 (1)	8.27	16.11
14	160 (0)	10 (−1)	0.05 (1)	4.58	27.99
15	160 (0)	10 (−1)	0 (−1)	27.85	9.80

**Table 2:** Regression coefficients and statistical parameters for model correlation and statistical significance.

Coefficient	Monosaccharides (g/L)	Oligosaccharides (g/L)
Intercept (b_0_)	36.39	2.30
Variable *x*_1_ (b_1_)	1.87	−7.95 ^a^
Variable *x*_2_ (b_2_)	0.06	−0.24
Variable *x*_3_ (b_3_)	4.19 ^c^	−5.19 ^b^
Variable *x*_1_*x*_2_ (b_12_)	−4.66	2.19
Variable *x*_1_*x*_3_ (b_3_)	−5.23	2.88
Variable *x*_2_*x*_3_ (b_3_)	−2.10	3.92 ^c^
Variable *x*_1_^2^ (b_11_)	−7.48 ^c^	2.52
Variable *x*_2_^2^ (b_22_)	−5.28	4.87 ^c^
Variable *x*_3_^2^ (b_33_)	−10.78 ^b^	10.10 ^a^
R^2^	86.77	94.56
Adjusted R^2^	62.97	84.76
Residual standard error (REE)	5.61	3.84
*F*-value	0.084	0.011

^a^ Coefficients significant at a 99% confidence level. ^b^ Coefficients significant at a 95% confidence level. ^c^ Coefficients significant at a 90% confidence level.

## Data Availability

The original contributions presented in this study are included in the article. Further inquiries can be directed to the corresponding authors.

## Supplementary Materials

The following supporting information can be downloaded at https://www.mdpi.com/article/10.3390/molecules31152654/s1, Figure S1: HPLC chromatograms for (a) Standards for glucose, xylose, galactose arabinose and fructose, (b) monophasic system, (c) monophasics posthydrolysis sample1 (d) monophasic posthydrolysis sample2 (e) biphasic system (f) biphasic posthydrolysis sample1 (g) biphasic posthydolysis sample2. Figure S2: HPSEC chromatograms for (a) standards (b) monophasic system (c) biphasic system.
