# Supplementary material for: Transforming Apple Pomace into Functional Oligosaccharides via Microwave Optimisation
Source: Molecules. 2026 Jul 30;31(15):2654. doi: 10.3390/molecules31152654 (PMC13468523; doi:10.3390/molecules31152654)
Supplement: Supplementary file 1 [file molecules-31-02654-s001.zip › molecules-4357042-supplementary.pdf]

Supplementary material

Transforming Apple Pomace into Functional Oligosaccharides via Microwaves Optimisation

Itsasne Beitia[1], Leyre Sillero[2], Itziar Egüés[1,*], Izaskun Dávila-Rodríguez[3,*]

[1]Biorefinery Processes Research Group, Department of Chemical and Environmental Engineering. University of the Basque Country UPV/EHU, Plaza Europa, 1, 20018, San Sebastián, Spain.

[2]Biorefinery Processes Research Group, Department of Chemical and Environmental Engineering. University of the Basque Country UPV/EHU, Nieves Cano, 12, 01006, Vitoria-Gasteiz, Spain.

[3]Biorefinery Processes Research Group, Department of Chemical and Environmental Engineering. University of the Basque Country UPV/EHU, Plaza Rafael Moreno "Pitxitxi" 2, 48013, Bilbao, Spain.

*Correspondence: itziar.egues@ehu.eus (I.E.); izaskun.davila@ehu.eus (I.D.-R.)

a)

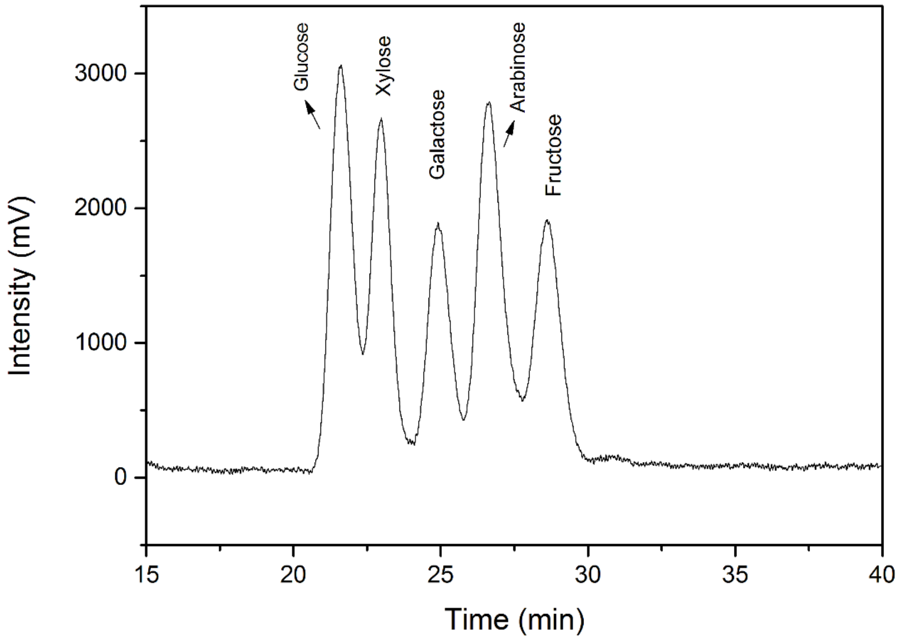
Glucose
Xylose
Galactose
Arabinose
Fructose
3000
2000
1000
0
Intensity (mV)
15
20
25
30
35
40
Time (min)

b)

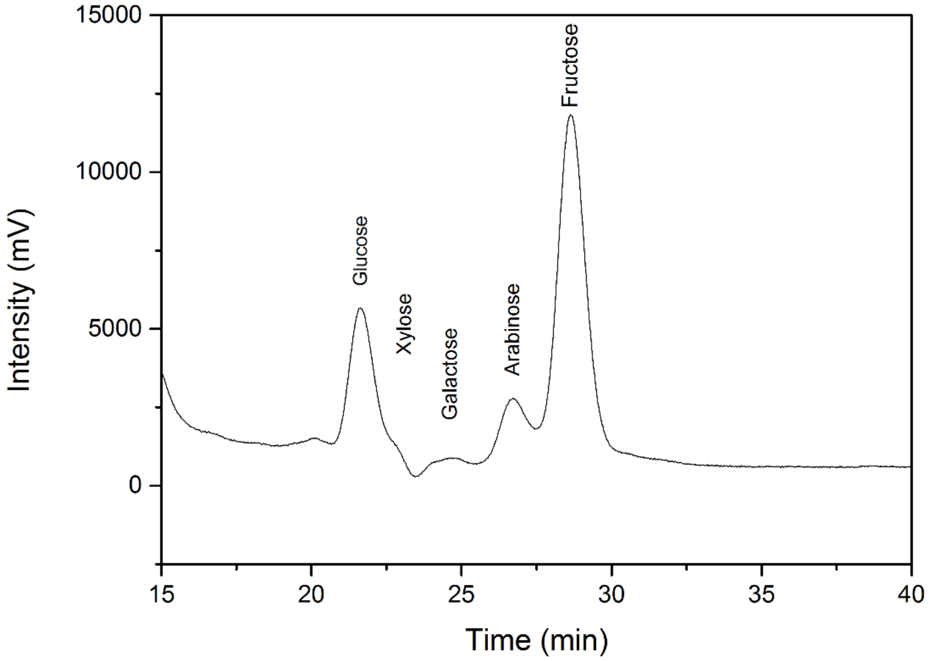
Fructose
Glucose
Xylose
Galactose
Arabinose
15000
10000
5000
0
Intensity (mV)
15
20
25
30
35
40
Time (min)

c)

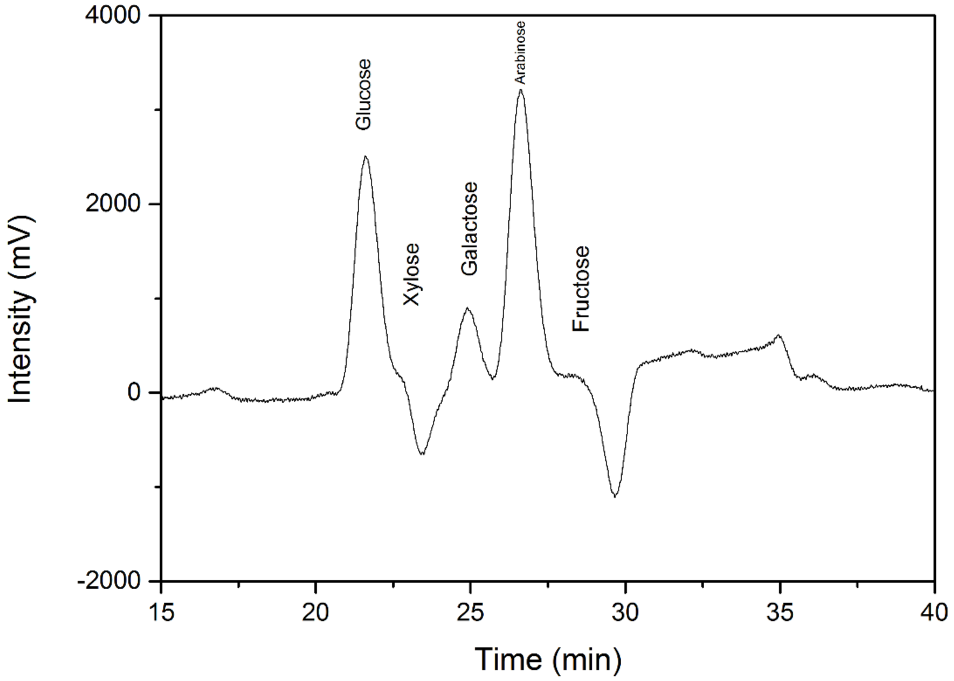

4000
2000
0
-2000
Intensity (mV)
15
20
25
30
35
40
Time (min)
Glucose
Xylose
Galactose
Arabinose
Fructose

d)

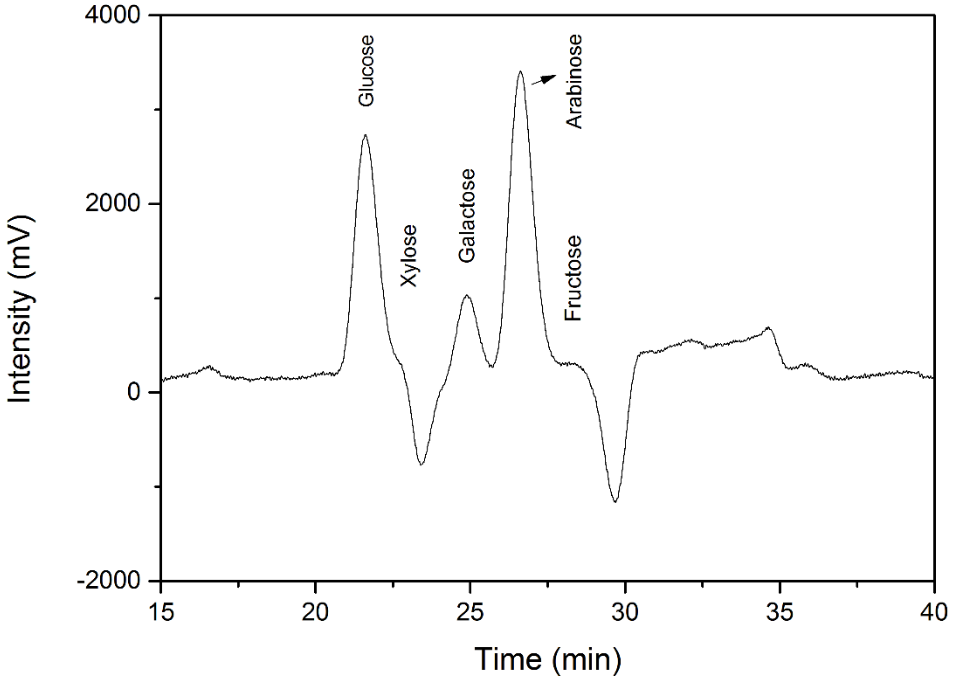

4000
2000
0
-2000
Intensity (mV)
15
20
25
30
35
40
Time (min)
Glucose
Xylose
Galactose
Arabinose
Fructose

e)

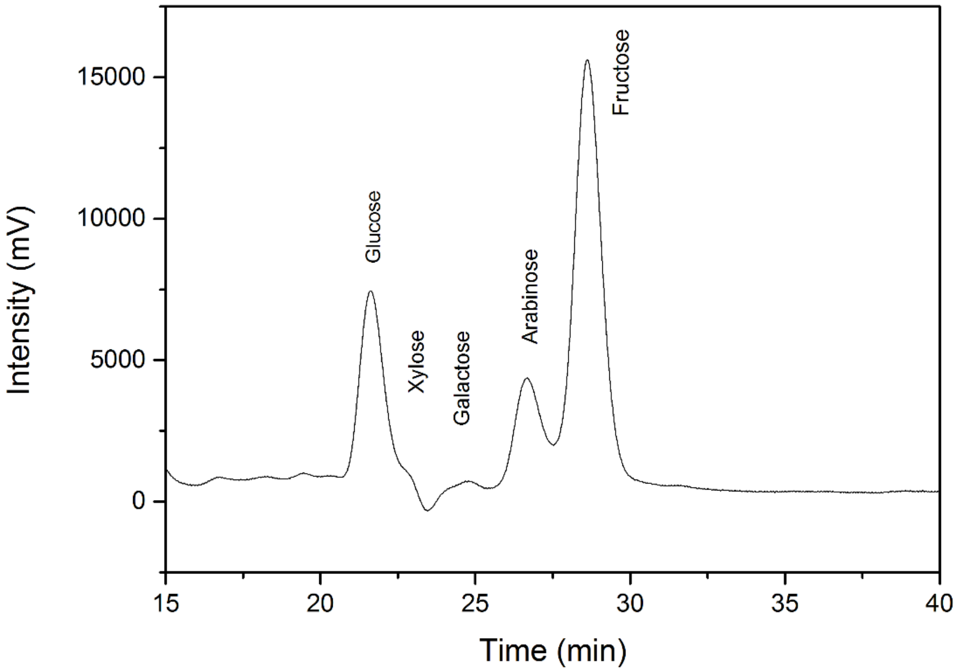
Intensity (mV)
Time (min)
Glucose
Xylose
Galactose
Arabinose
Fructose
15000
10000
5000
0
15
20
25
30
35
40

f)

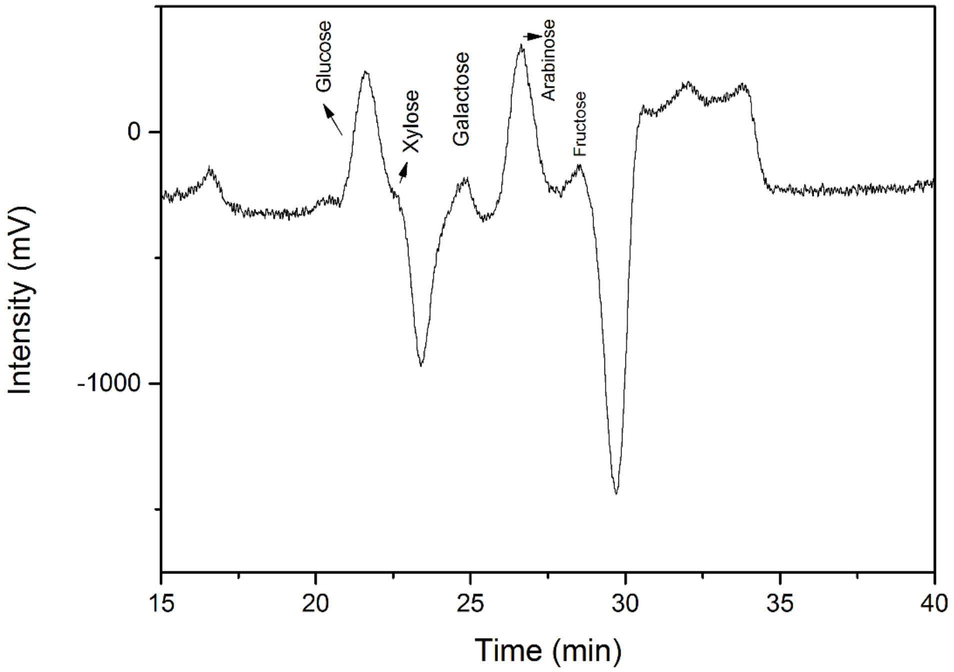
Intensity (mV)
Time (min)
Glucose
Xylose
Galactose
Arabinose
Fructose
0
-1000
15
20
25
30
35
40

g)

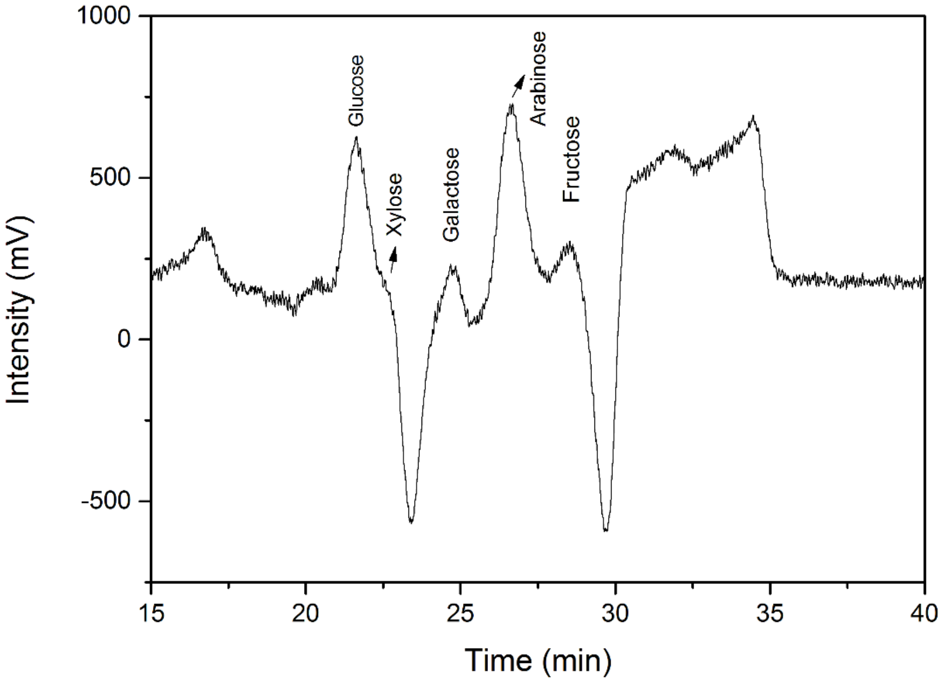

Figure S1. HPLC chromatograms for a) Standards for glucose, xylose, galactose arabinose and fructose, b) monophasic system, c) monophasics posthydrolysis sample1 d) monophasic posthydrolysis sample2 e) biphasic system f) biphasic posthydrolysis sample1 g) biphasic posthydolysis sample2.

a)

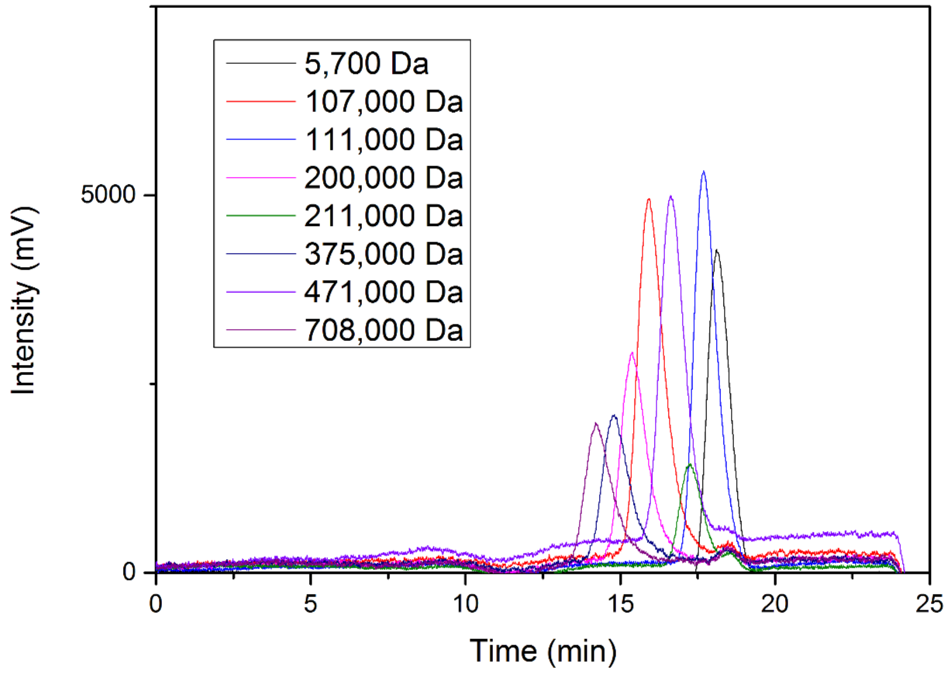
5,700 Da
107,000 Da
111,000 Da
200,000 Da
211,000 Da
375,000 Da
471,000 Da
708,000 Da
Intensity (mV)
5000
0
0
5
10
15
20
25
Time (min)

b)

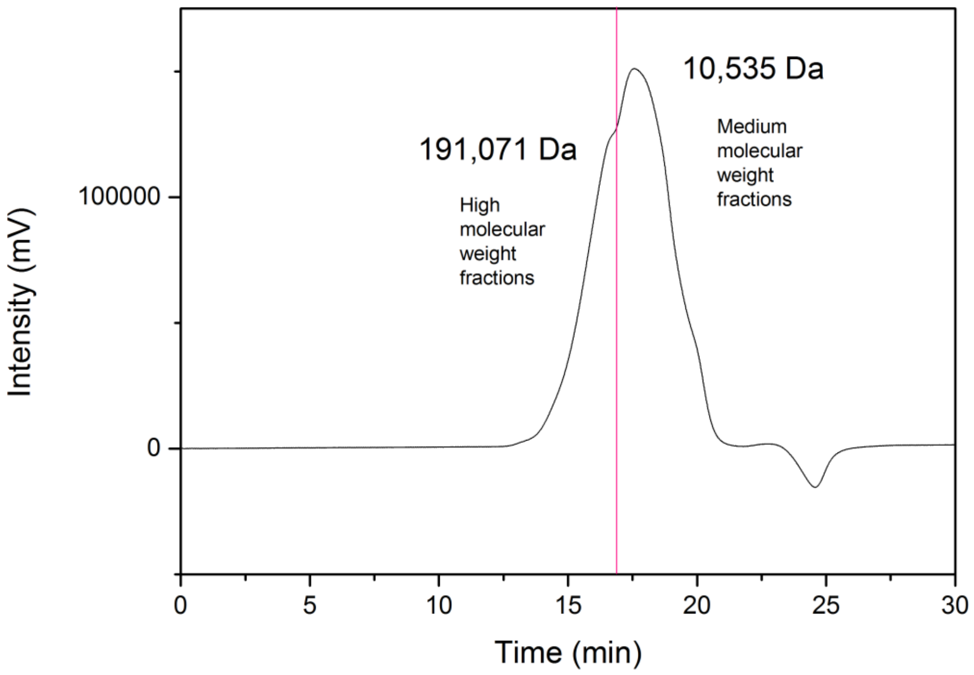
191,071 Da
High molecular weight fractions
10,535 Da
Medium molecular weight fractions
Intensity (mV)
100000
0
0
5
10
15
20
25
30
Time (min)

c)

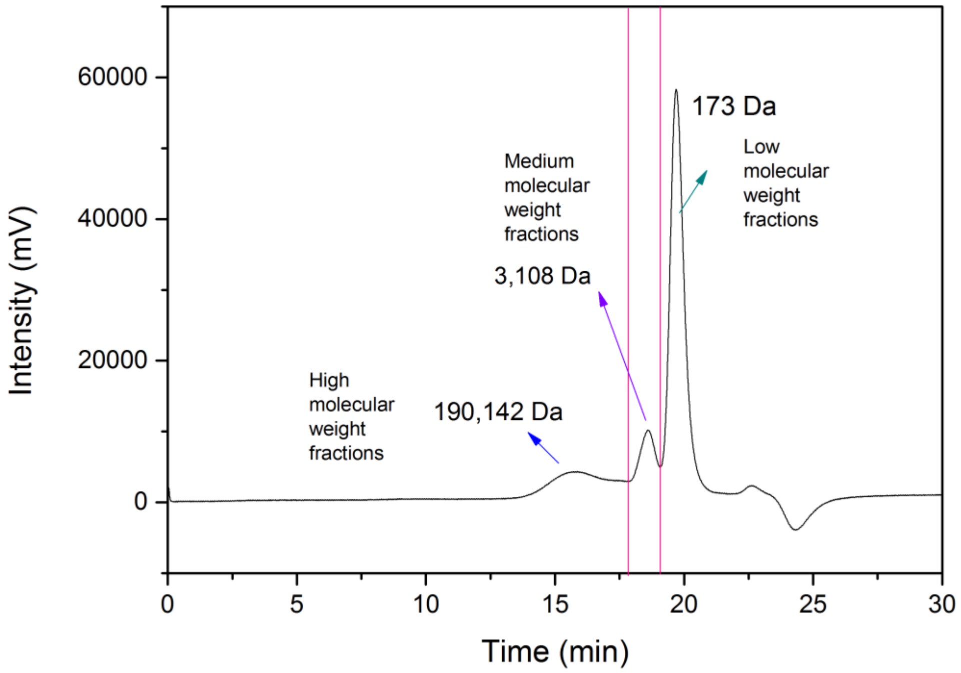

Figure S2. HPSEC chromatograms for a) standards b) monophasic system c) biphasic system.
